# Societal Risk Evaluation Scheme (SRES): Scenario-Based Multi-Criteria Evaluation of Synthetic Biology Applications

**DOI:** 10.1371/journal.pone.0168564

**Published:** 2017-01-04

**Authors:** Christopher L. Cummings, Jennifer Kuzma

**Affiliations:** 1 Wee Kim Wee School of Communication and Information, Nanyang Technological University, Singapore; 2 School of Public and International Affairs, North Carolina State University, Raleigh, North Carolina, United States; US Army Engineer Research and Development Center, UNITED STATES

## Abstract

Synthetic biology (SB) applies engineering principles to biology for the construction of novel biological systems designed for useful purposes. From an oversight perspective, SB products come with significant uncertainty. Yet there is a need to anticipate and prepare for SB applications before deployment. This study develops a Societal Risk Evaluation Scheme (SRES) in order to advance methods for anticipatory governance of emerging technologies such as SB. The SRES is based upon societal risk factors that were identified as important through a policy Delphi study. These factors range from those associated with traditional risk assessment, such as health and environmental consequences, to broader features of risk such as those associated with reversibility, manageability, anticipated levels of public concern, and uncertainty. A multi-disciplinary panel with diverse perspectives and affiliations assessed four case studies of SB using the SRES. Rankings of the SRES components are compared within and across the case studies. From these comparisons, we found levels of controllability and familiarity associated with the cases to be important for overall SRES rankings. From a theoretical standpoint, this study illustrates the applicability of the psychometric paradigm to evaluating SB cases. In addition, our paper describes how the SRES can be incorporated into anticipatory governance models as a screening tool to prioritize research, information collection, and dialogue in the face of the limited capacity of governance systems. To our knowledge, this is the first study to elicit data on specific cases of SB with the goal of developing theory and tools for risk governance.

## Introduction

Synthetic biology (SB) seeks to identify and apply principles of biology in the design of biological parts and systems in order to create and redesign natural biological systems for useful purposes [1]. SB is expected to provide benefits to society in multiple sectors in a bioeconomy including agriculture and food production, bioenergy, biosensor development, chemical synthesis, environmental protection and remediation, and human health among others. However, it is also likely to pose risks and societal impacts that are undesirable depending on the project and systems in which its applications are deployed [2]. While most SB applications are being developed for their extrinsic benefits to society, these same technologies may create human and environmental hazards. Generally, microorganisms exist and interact within a highly complex environment and they derive their genetic functions from evolutionary processes that occur over millennia of trial and error. Biologists and engineers working in SB are making evolutionary leaps through time as they craft highly engineered organisms capable of researcher-specified responses to stimuli. Such advancements hold promises to make significant impact on society through beneficial applications, but may also create concern for harmful mishaps, misuses, or unintended consequences, and disrupt ecosystem functioning.

The current era of scientific and technological development promises that SB will provide us with the knowledge and skill to engineer living systems, and to potentially alter natural evolutionary processes. In recent history the engineering of organisms and deployment into agriculture (e.g. GM crops) has caused a great deal of controversy, including the call for bans and labelling, trade disruptions, and scientific disputes [3, 4, 5]. Some scholars contend that thoughtful public exploration of the effects of GMOs prior to widespread adoption has been absent; others have argued that a similar lack of transparency regarding decision-making has arisen, and that socioeconomic and ethical issues are often dismissed [6, 7]. This context motivated us to assess oversight and governance structures “upstream” of widespread SB deployment and use—in an earnest effort to anticipate possible health, environmental, and social impacts. This work extends traditional assessment of dosage and exposure estimates with a novel methodological contribution that supports a new rubric for risk assessment that anticipates health, environmental, and social impacts of emerging technologies along with more traditional risk assessment measures.

Calls for anticipatory governance for biotechnology and nanotechnology have emerged in the past two decades [8, 9]. Anticipatory governance uses three principles: 1) foresight, 2) the integration of natural and social science research, and 3) upstream public engagement [10]. Upstream public engagement has also been proposed to open the dialogue of emerging technological products to wider publics [11]. However, the applications of SB are numerous and to discuss it as a whole with respect to governance is often not productive, as the risks and benefits, ethical challenges, and other societal aspects vary significantly by product. As a result, scholars have called for analyses of the issues and appropriate governance regimes on a case by case basis [12, 13]. Although there are distinguishing features of synthetic biology as a whole, case studies are useful for deeper conversations about risks, benefits, socioeconomic aspects and ethical issues.

Another concept in the literature, “prevention-focused governance” [14] aligns with anticipatory governance but also suggests the use of multi-criteria decision analysis (MCDA) to support risk governance of emerging technologies through a multi-criteria assessment of the trade-offs of the technology prototype compared to existing alternatives [15]. MCDA involves the use of methods and tools to measure and integrate several criteria into decision making. These criteria can be derived and measured by using expert or stakeholder judgement in order to assess technology cases or prototypes in development and upstream of deployment [16]. Based on the MCDA, preventative governance then asks whether the emerging technology prototype should be developed when compared to other existing options to address the problem [14]. Here we extend MCDA approaches and develop a tool to screen projected applications of synthetic biology in order to anticipate and prepare for risk governance, and if necessary, prevent situations of unacceptable adverse impacts.

Our previous work applied multiple criteria to emerging cases of nanotechnology applied to food and agriculture while focusing more on risk-based and oversight policy choices with which decision-makers are faced. We termed this approach “upstream oversight assessment” (UOA), as a subset of anticipatory governance [17, 18]. UOA is distinct from other types of technology forecasting and assessment in its approach and focus on oversight systems, yet it shares properties of participation, anticipation, interdisciplinarity, integrated fact and value perspectives, and its policy studies orientation to inform a better future [19]. UOA involves the selection and analysis of case studies upstream of deployment and in early development stages in order to inform wider systemic risk governance issues as well as specific product issues [17]. In this previous work, broad criteria were used in a MCDA approach and were qualitatively assessed using existing literature in order to highlight areas needed for future regulation and decision making.

This paper extends UOA and MCDA approaches to anticipate and prepare for SB governance through a scenario-based evaluation of four distinct SB applications. We use a multidisciplinary expert Delphi study to develop and evaluate criteria important for informing risk governance of SB applications that are in early development. Criteria were developed to focus on informing risk governance for environmental releases of the SB applications and were quantitatively assessed through expert and stakeholder judgement. These extend the risk analysis paradigm and traditional MCDA approaches beyond direct human and environmental risks and benefits by including important criteria in the face of significant uncertainty, such as reversibility and manageability, which are particularly important for SB.

The study incorporated a panel of experts from various disciplinary backgrounds working in SB and posed the following broad research questions which framed the creation of the SRES:

1. How can societal impacts of SB technologies be incorporated with traditional EHS estimates to better inform decision-makers?
2. How can uncertainty of information, and confidence in expert judgements be incorporated into risk and benefit estimates of SB technologies?

With these two research questions in mind, we conducted a four-round Policy Delphi study that culminates in the novel 8-component Societal Risk Evaluation Scheme (SRES) that incorporates hazard and exposure estimates, social impacts, and multiple forms of uncertainty estimates. It is inspired by the RES work of Latxague et al. (2007) [20] and Suffert et al (2009) [21], while adding factors explicit for SB and including more than direct human and environmental harm, such as likelihood of commercialization and public concern. We therefore term our approach Societal and Risk Evaluation Scheme (SRES). Besides the broad research questions, this study also addresses the following questions regarding practical decision-making, methods and process, and theory-building:

1. Practical decision making: What are the potential areas of concern or impact associated with the SB applications What are the associated uncertainties?
2. Methods and process: What methods for anticipatory risk governance could be used in the face of significant uncertainties? How might they be integrated into wider stakeholder deliberation and assessment models? How can they account for multiple types of impacts or considerations?
3. Theory-building: What features of the technology affect risk and technological perceptions of diverse subject matter experts (SMEs)?

In this paper, the last question was informed by the psychometric paradigm for risk perception and technology acceptance [22, 23]. Features of the applications were typed according to the psychometric paradigm’s scales of known/unknown, controllable/uncontrollable, etc. and the panel rankings of the cases compared to these features to see if larger conclusions could be drawn. Our findings contribute theoretically to the psychometric paradigm, noting that issues of controllability and more specifically uncontrollability are likely to play a significant role in the assessment and potential use of the SB technologies assessed in this project.

In summary, we present here a new analysis of SB cases with practical policy-making, methodological, and theoretical contributions. The ranking of SB cases using the SRES framework allows for preparation for oversight of specific areas of SB, while cross-case comparisons help to derive general lessons or hypotheses broadly associated with priorities for broader types of applications and risk perception theory. We present our methodology and results below and then discuss how the SRES could be integrated into broader societal conversations about SB and other emerging technologies.

Of note, we anticipate that data provided in this paper should be used with caution for current decision-making regarding SB technologies. This work supports adapting current risk assessment schema to include greater emphasis on social risks and uncertainty that are drivers of public acceptance, future use, and governance of emerging technologies.

## Methodology

While UOA, Delphi studies, and RES are all established methods, this novel study furthers methodological contributions to the field through the use of expert-only derived data. Previous RES work relied on subjective coding of secondary sources, which may be suspect to observational biases. The Delphi method described in full below curtails such researcher biases that may have been present in previous RES work, and seeks to maximize objectivity of the reported findings regarding the SB applications. This method of data collection allows for greater internal validity of findings in the SRES model than previous researcher-derived RES data models. The work reported here is situated within a larger project titled “Looking Forward to Synthetic Biology Governance: Convergent Research Cases to Promote Policy-Making and Dialogue” which was funded by the Alfred P. Sloan Foundation. The grant employed a four-round Policy Delphi method to facilitate data and information collection using a multidisciplinary expert panel. Methods of future studies and policy sciences were used to develop and examine cases of SB in the context of risk policy and governance [24, 25]. In particular, UOA was used to help select cases, develop initial drafts of the case studies, and construct questions to inform risk analysis and oversight policy [26].

The Policy Delphi process [27] was used to refine the case studies and elicit expert-stakeholder opinions about the potential risks, benefits, and ethical, legal, and societal (ELSI) issues associated with the SB cases. Named after the Oracle of Delphi, the Delphi method is designed to maintain group dynamics of small expert panels to distil responses and build toward group consensus—although this is not a requirement of some forms of the Delphi as will be further illuminated below. The method is typically used “when accurate information is unavailable or expensive to obtain, or evaluation models require subjective inputs to the point where they become the dominating parameters” [27]. The Delphi method is inclusive, but also allows respondents to voice themselves anonymously and enables panel members to change their opinion without fear of repercussion. Experts respond individually and on their own time-scale within multiple rounds of inquiry. The Delphi method procedurally blends polling and conferencing but unlike conference telephone calling and formal seminars, these studies force delays allowing a study’s administrators to maintain “equal flow of information” to and from all panel members and granting each member time to reflect on issues within the study. The Policy Delphi is therefore a tool for the analysis of policy issues and not a mechanism for making a decision [28]. Our goal for this study was to employ the Policy Delphi method to investigate societal impacts and uncertainty along with risk and benefit analyses.

This Delphi consisted of four rounds. The first round consisted of a standardized open-ended interview, which is a form of qualitative data collection that is more structured than most other interview methodologies and thus “increases comparability of responses” [29]. The second round was comprised of an online quantitative survey that was created from qualitative content analyses using the constant comparative method of the interview data in Round One [30]. Within the survey, panel members were asked to respond to scale items regarding a variety of risk and governance issues detailed in the following section of this paper. 35 participants completed the second round of the study, but one case was removed from data analysis as is detailed below. The third round consisted of a face-to-face workshop where the goal was to envision ideal governance for SB in coming generations. The final round consisted of another shorter online survey used to assess general trends in expert opinions of important factors that might contribute to future governance schemes based on SB application types.

This paper largely describes round two, the first survey which built upon the interviews to formulate questions of risk analysis and governance, and also provides qualitative findings and quotations from the first round of interviews. Prior to the interviews and survey, the participants were asked to read four short case studies about synthetic biology applications (see Appendix A). These cases and the case-study approach built upon previous examination of SB’s pernicious governance profile outlined by Kuzma and Tanji (2010) who note that “different categories of SB application may warrant different oversight regimes, and there might not be an appropriate ‘one size fits all’ approach…and we argue that policy recommendations should be built from consultation with experts and stakeholder multiple disciplines, and developed in the presence of stakeholders and public citizens” [2]. This project takes up the call of a more robust policy analytical approach of various SB application fields through the use of case studies within the Policy Delphi rubric. Findings from other rounds of the Delphi will be reported in other publications.

### Case study selection

In selecting our case studies, we first identified and reviewed popular media reports on synthetic biology research, cases that were subjects of other policy conversations, and the peer-reviewed and gray literatures on SB. Ten potential case studies were developed (2 pages to describe the SB technology, problem, and application) and sent to an internal panel at the researchers’ university comprised of synthetic biologists and policy scholars for review and comment about those that would best 1) cover a range of environmental release applications, 2) represent a range of SB technologies from highly engineered organisms to completely synthetic machines, and 3) show plausible cases that were being researched in labs at early or mid-stages or in other words, if successful would be deployed in 10 to 50 years. The final four used throughout the Policy Delphi project were: biomining using highly engineered microbes *in situ*, cyberplasm for environmental detection, de-extinction of the passenger pigeon, and engineered plant microbes to fix nitrogen on non-legumes.

A case study analysis approach guided by UOA and anticipatory governance was used to guide question formulation for the interview and survey protocols that would be used to analyze risk analysis and oversight policy issues [26]. Details on the expert sample, the Policy Delphi method, and the measures used to create the novel SRES are described below.

### The expert sample

The sampling of experts was done purposively in order to attain information on issues surrounding risk, governance and policy issues of applications of synthetic biology. Sampled experts represent “information rich” cases [31] that can provide greater insight into emerging SB issues than most potential panel members. Panel members were sought after from a range of disciplines associated with SB research areas in order to better address the complex milieu of issues to be addressed in the Policy Delphi project.

The list of potential panel members was compiled using a mixture of web database searches, literature reviews for relevant author names, and prior knowledge and experience by the research team. The initial set of experts contained 234 persons originating from a variety of groups including editorial review boards for SB research journals, attendance lists from research conferences and national and international professional association meetings, and government listed panels of research, policy, and ethics review committees. Few potential panel members were also added to the original listing based on personal review from other SB researchers and analysts as well as the research team. The goal was to recruit a well-balanced group of 50 experts to participate in the interview phase. Potential participants were emailed a recruitment letter and a survey sheet to self-identify their expertise and affiliation. Positive responses were tallied according to expertise, affiliation and bias (i.e. pro-SB technology development, to more critical of SB development or precautious about it). Additional participants were contacted in areas lacking appropriate representation to balance the group as much as possible. In total, 48 experts responded as willing to participate in the study and completed the interview phase. These 48 were invited to participate in all rounds of this Delphi study. However, not all participants were able to complete each round of the Delphi. Thirty-five members fully completed the survey used for the SRES framework. All participants were provided written and verbal informed consent prior to participation in this study. Participants were notified in email and via telephone that their participation in the study would serve as their knowledgeable consent. IRB approval for this study was granted through North Carolina State University, and all recorded information was stored under password protection and behind lock and key by the researchers. A list of the disciplines and affiliations of these experts is provided below (Table 1).

**Table 1:** Expert Panel by Discipline and Affiliation.

	Discipline	Affiliation
1	Science, Tech & Society	Academe
2	Chem/Mol/Bio Engineering	Academe
3	Chem/Mol/Bio/Phy/Math Engineering	Academe
4	Policy/Governance	Academe
5	Policy/Governance; Ecology/Environ Science	Academe
6	Sociology/Philosophy/Ethics	Academe
7	Policy/Governance; Science, Tech, and Society	Academe
8	Chem/Mol/Bio Engineering	Academe
9	Chem/Mol/Bio Engineering	Academe
10	Sociology/Philosophy/Ethics	Academe
11	Sociology/Philosophy/Ethics	Academe
12	Chem/Mol/Bio Engineering; Science, Tech & Society; Policy/Governance	Academe
13	Sociology/Philosophy/Ethics	Academe
14	Policy/Governance/Law	Academe
15	Policy/Governance	Academe
16	Chem/Mol/Bio Engineering; Science, Tech & Society; Ecology/Environmental Science	Academe
17	Science, Tech & Society; Policy/Governance	Academe
18	Policy/Governance	Government
19	Ecology/Environ Science	Government
20	Chem/Mol/Bio Engineering; Science, Tech & Society	Government
21	Ecology/Environ Science	Government
22	Ecology/Environ Science	Government
23	Policy/Governance	Government
24	Chem/Mol/Bio Engineering	Government
25	Policy/Governance; Human Health/Toxicology/Epidemiology	Government
26	Chem/Mol/Bio Engineering	Industry
27	Chem/Mol/Bio Engineering	Industry
28	Science, Tech & Society	Industry
29	Chem/Mol/Bio Engineering	Industry
30	Chem/Mol/Bio Engineering	Industry
31	Policy/Governance	NGO
32	Ecology/Environ Science	NGO
33	Law	NGO
34	Science, Tech & Society; Policy/Governance	NGO
35	Policy/Governance	NGO

The final panel that completed the SRES survey used in this article was comprised of experts who self-identified as chemists, molecular biologists, engineers, sociologists, toxicologist, environmental scientists, lawyers, STS scholars, philosophers, and policy practitioners or scholars, and others. Several experts identified with more than one disciplinary group. Although balance was the goal, the group was limited to those experts willing to participate in the survey and therefore is self-selected in this regard. Although broad range of disciplines and affiliations appear on the panel group, each discipline is not equally represented. Expert elicitation is not meant to be a representative methodology, and for the broad area of technology governance, it is difficult to include equally all relevant disciplines and expertise areas. In our case, one out of every five experts were from ecology/ environmental sciences/ toxicology; whereas about one out of every three had expertise in chemistry/molecular biology/bioengineering. The lower number of ecology/environmental science/toxicology experts who were successfully recruited for the study could be due to fewer experts overall in these areas with respect to SB, as funding for studies on the health and environmental safety of synthetic biology is very small relative to funding for technology development. Also, about half of the experts were from academe, five from NGOs, eight from government, and six from industry which might reflect the early emergence of the field of SB out of academe.

### Survey measures

Findings presented forthwith come from data collected in the second stage of the larger Policy Delphi project funded by the Alfred P. Sloan Foundation. The first round involved a standardized open-ended qualitative interview used to highlight pressing areas and issues concerning risk governance and data needs, and the second round employed a quantitative online survey using Qualtrics to populate the data for this paper. The survey also included open-ended questions to develop questions for rounds 3 and 4 of the Delphi (not analyzed here).

Data were collected among all expert respondents for each of the survey measures. Following listwise deletion of respondents who answered less than 50% of the survey items [32] the final sample was (N = 34). The number of participants in each round is shown below (Table 2). As previously noted, survey measures were designed to account for multiple facets of risk evaluation and are further detailed below.

**Table 2:** Expert Participants by Delphi Rounds.

Round	Type	Participants	Use in this paper
1	standardized open-ended interview	N = 45	Supplement for discussion
2	Online survey including SRES scales and open-ended questions	N = 34	Primary data source for SRES
3	Workshop survey of ideal governance characteristics	N = 35	Supplement for discussion
4	Online survey of future governance schemes	N = 35	Supplement for discussion

The SRES includes 8 sections evaluated using a 0 to 9 semantic differential integer scale The SRES survey items are inspired from the work of Suffert et al. (2009) and Latxague et al. (2007) who developed an RES for case studies of agricultural pathogens used in bioterrorism [20, 21]. Their original work was based out of their subjective content analytic coding of findings from peer-reviewed journal articles. They produced pentagonal star plots that detailed the risks of nine plant pathogens based upon 5 criteria; ease of use of the pathogen, importance of the target crop, epidemic potential of the pathogen, obstacles to swift and effective response, and potential global or regional consequences. We adapted their criteria to better reflect the UOA governance needs of these emerging synthetic biology applications. Each of the measures used in the SRES stemmed from preliminary textual analysis of the qualitative interviews from Round One of the Policy Delphi. Also, our project embedded the SRES in the larger Delphi process using expert elicitation, whereas previous work relied on the author’s judgements of published literature. Our expert panel of respondents provided all the information used within our SRES models and there was no reliance on subjective estimation on our part as to where secondary textual sources would fall on a scale measure.

The SRES involves a broader conception of risk than the strict “technical” definition of the severity of the hazard combined with the likelihood of occurrence or exposure. We call it a “societal risk” evaluation scheme as it includes psychometric and social factors that affect how people perceive risk and what they place value upon in preventing, mitigating, or accepting risk Specifically we asked the expert panel to respond to eight criteria including: R1) potential hazard to human health, R2) potential hazard to the environment, R3) degree of unmanageability of the potential hazards, R4) degree of irreversibility of the potential hazards, R5) likelihood of commercial development in the next 15 years, R6) potential benefit to the human health, R7) potential benefit to the environment, and R8) degree of public concern. The table below (Table 3) details the exact survey items and semantic endpoints used in the questionnaire. Each of the SRES criteria was measured using a 0–9 integer semantic differential scale. In order to better facilitate graphical representation of the SRES data, all terms were phrased to highlight their higher risk condition. For instance, criteria R6 “potential benefit to human health” was reverse coded and phrased as “lack of benefit to human health.”

**Table 3:** SRES Survey Items.

Survey Item	Semantic Differential Scale Endpoints
R1: How potentially hazardous is [SB APPLICATION] to human health?	0 = Completely unhazardous; 9 = Completely hazardous
U1: How confident are you about your answer to the previous question?	0 = Completely unconfident; 9 = Completely confident
R2: How potentially hazardous is [SB APPLICATION] to the environment?	0 = Completely unhazardous; 9 = Completely hazardous
U2: How confident are you about your answer to the previous question?	0 = Completely unconfident; 9 = Completely confident
R3: How manageable are the potential hazards of [SB APPLICATION] (reverse coded)?	0 = Completely manageable; 9 = Completely unmanageable
U3: How confident are you about your answer to the previous question?	0 = Completely unconfident; 9 = Completely confident
R4: To what degree are the potential hazards of [SB APPLICATION] irreversible?	0 = Completely unlikely; 9 = Completely likely
U4: How confident are you about your answer to the previous question?	0 = Completely unconfident; 9 = Completely confident
R5: How likely is [SB APPLICATION] to be commercially developed and used in the next 15 years?	0 = Completely unlikely; 9 = Completely likely
U5: How confident are you about your answer to the previous question?	0 = Completely unconfident; 9 = Completely confident
R6: How beneficial is [SB APPLICATION] to human health (reverse coded)?	0 = Completely not beneficial; 9 = Completely beneficial
U6: How confident are you about your answer to the previous question?	0 = Completely unconfident; 9 = Completely confident
R7: How beneficial is [SB APPLICATION] to the environment (reverse coded)?	0 = Completely not beneficial; 9 = Completely beneficial
U7: How confident are you about your answer to the previous question?	0 = Completely unconfident; 9 = Completely confident
R8: What might be the level of public concern regarding the risks of [SB APPLICATION]?	0 = Completely unconcerned; 9 = Completely concerned
U8: How confident are you about your answer to the previous question?	0 = Completely unconfident; 9 = Completely confident

These criteria have strong roots in social scientific investigations of risk as well as from the social and physical sciences. For example, unmanageability and irreversibility are included in the SRES as psychometric risk perception factors [22]. Benefits and overall “concern” about technology (affect) also strongly influence risk perception and public attitudes towards new technologies (reviewed in [4]) and are also included in the SRES. Our SRES framework includes toxicological and epidemiological, economic, technological, psychometric, and social approaches to the conception and assessment of risk as described in Renn’s 1992 work [33]. Each of the 8 of sections of the SRES was plotted on the circumradials of an octagon that was then overlaid with a second circumradial dial of uncertainty measures associated with each of the 8 sections. Each spoke of the octagon corresponds to the SRES measures with the center-point being equal to a score of zero on each measure. The second circumradial dial of uncertainty was created from questionnaire items that asked experts to provide their level of confidence associated with each factor. We chose to highlight this area as the evaluation of uncertainty is paramount to risk assessment throughout the assessment process. Uncertainty can be manifested when there are conflicting, inconsistent, incomplete, and even missing data, or due to the subjectivity of analysis based upon the risk assessor [34, 35]. These measures provide expert estimates of uncertainty via confidence regarding the current state of data and information of each section of the SRES. Confidence reports also help to assess the overall uncertainty associated with the current state of understanding for each SB application. Identification of areas of uncertainty and transparent communication of such areas is vital for stakeholders and decision-makers alike and should be accounted for when prioritizing future research options and data needs.

In order to quantify such vital information regarding uncertainty levels of each case, a corollary battery of survey measures was used to assess each of the eight sections of the SRES and was also judged on a [0–9] semantic differential integer scale ranging from “completely unconfident” to “completely confident”. Individual item uncertainty is reported below as well as an overall uncertainty estimate of the risks of each of the four cases in the final section of the SRES. Data were catalogued and analyzed using IBM SPSS 20. The few cases of missing data (less than 2%) were transformed using the linear trend at point function of SPSS that replaces missing values with the linear trend for that data point where the existing series is regressed on each indexed variable scaled 1 to *n* and each missing value is replaced with its predicted value giving the regression line. Given the objective of this work to be among the first to synthesize findings across expert fields in order to guide practical decision-making, we felt it appropriate to not split our limited sample size into subject matter groups and instead provide whole panel data that characterizes the entire sample’s responses.

The resulting octagonal plots were designed as single visual representations of the multidimensional risk profiles of each SB application under investigation. While the components of the case studies and the SRES measures are non-exhaustive, the risk profiles, which quantify the risk associated with each potential SB application, provide heuristic value to promote governance dialogue and improve decision-making regarding upstream governance and decision making regarding data needs for future risk assessment.

Each SB application is summarized on the SRES using an additive risk index and ultimately a mean risk score (*R*):

$$R=(\sum_{i=1}r_{i})/n$$

with *r*_*i*_ is the *i*th variable of the SRES and n = 8. Similar uncertainty scores (*U*) were calculated to better inform stakeholders and risk assessors.

## Results & Discussion

From a practical perspective, this study develops a new SRES framework designed to be used in anticipatory governance and upstream oversight assessment when little data or information is available to help with prioritization of future data and information collection agendas. The SRES may provide heuristic function to highlight areas of need concerning risk and benefit evaluation, societal impacts, and uncertainty of current information.

From a theoretical perspective, the psychometric paradigm of risk perception [22, 23] was used to see if factors related to the case studies themselves accounted for the risk rankings of the experts. For this, the case study SB applications were typed according across the two primary grid factors in psychometric risk perception theory. This typing was first done by the research team based on the features of the cases themselves. The panel rankings of the cases were compared to what one would predict from the psychometric paradigm. Fit with the paradigm and discrepancies are discussed in light of the rankings of the other SRES measures outside of psychometric factors from Slovic (1987) [22] that broaden the analysis, as well as of other societal implications of the cases identified from the interviews.

The section is organized as follows: 1) first, we give a brief description of each case study, type them according to the psychometric grid and discuss how we would expect the cases to be ranked from psychometric theory, 2) we then describe how the risks, uncertainties, benefits, and feasibility of each case were scored in the SRES, highlighting differences and similarities among the cases, 3) next, we ask whether the psychometric paradigm held true for the quantitative SRES rankings of the cases that relate to the theory, 4) then, we discuss additional factors in the interviews and SRES (those outside of psychometric theory) that might also explain discrepancies in the risk ranking, and 5) finally, from a practical motivation, we illustrate how priority areas can be derived from the SRES scores and the interviews to inform anticipatory governance processes.

### Case Description and Typology

The four case studies used in the research were derived from expert input and review as to a set that would represent a range of SB applications in industry, agriculture, and the environment. We focused on medium to longer term cases of SB, as opposed to ones already on the market which largely only involve the addition of a few genes or pathways using genetic engineering, in order to demonstrate a methodology that could be used in anticipatory governance processes under conditions of high uncertainty and that could be deployed prior to market entry. Two of the four cases were chosen to represent medium-term applications of SB. For biomining and nitrogen fixation, genetically engineered products are already in design and deployment phases and SB extends the genetic engineering methods used in these applications. The two other cases, de-extinction and cyberplasm, were chosen to represent applications of SB further out into the future. The set of cases was designed to illustrate how the SRES framework could guide risk governance for applications of emerging technologies prior to market entry in upstream assessments and upstream public dialogues.

**Fig 1:**
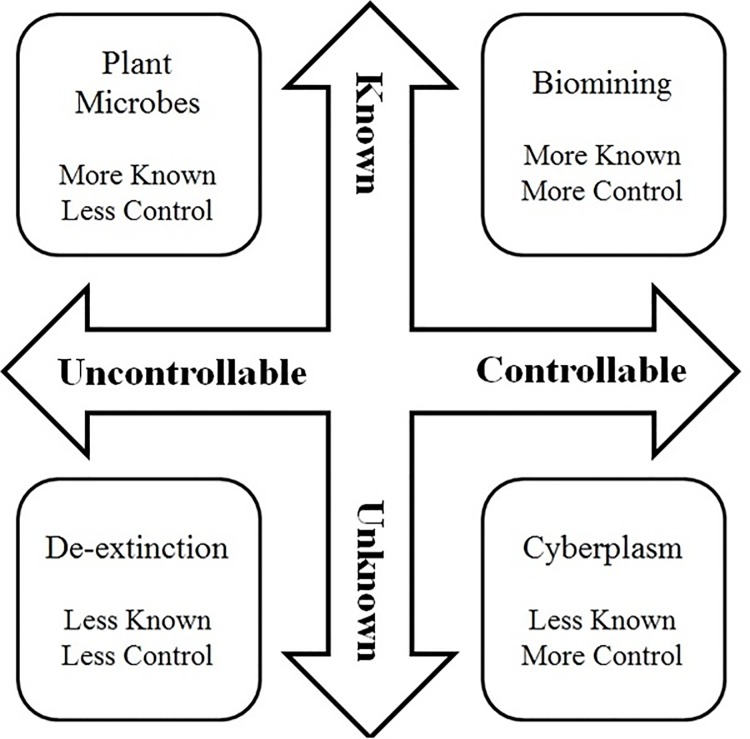
Visual Representation of Slovic’s (1987) Known/Controllable Dimensions of Risk

Two page descriptions were sent to the expert group prior to the interviews and survey rounds. First, cases were described in general terms, then specific example(s) were given, and the general concerns and benefits were mentioned with the intent of balance on the part of the study team. Fig 1 depicts the grid based on Slovic (1987) for the 4 case studies, and rationales for project team typing of the cases is described below.

The biomining case was described in general as engineered bacteria used to extract minerals from the soil *in situ*, with the specific example of engineered *A*. *ferroxidans* to yield enhanced characteristics in differentiation, memory, and pattern forming that would enhance metal extraction [36]. This case study was typed as “Known/Controllable” on the two scale grid described by Slovic (1987), because similar bacteria have been used (without the engineered traits) and the deployers of the technology would largely have control over the release and reclamation of the bacteria (although residual spread could occur). Several companies are using non-engineered bacteria for biomining, and a few are developing highly engineered bacteria for it [37].

The cyberplasm case was described as a “convergence of synthetic biology, biomimicry, nanotechnology, and robotics to construct a micro-scale robot designed to be capable of sensing and treating pathogens within plants and animals or for other functions involving sensing and remediation”. It was chosen as a true example of a synthetic organism and because it was funded and is in development, but further away from deployment than the others. Uses of the micro-robot were described for healthcare and environmental remediation, and the parts were described as coming from bacteria, yeast, and mammalian cells [38]. This case study was typed by the project team as “Unknown/Controllable” for its very high degree of novelty, but inability of the robots to self-replicate (like living genetically engineered organisms) and settings in which they deployed.

De-extinction was used as a feasible, longer term case study of synthetic biology and described as the bringing back of certain species into the environment that have become extinct (largely because of human interventions). Examples of species for which researchers have been somewhat successful in using cloning or de novo DNA synthesis or editing for this purpose were described: a gastric brooding frog and a wild mountain goat. Current efforts in academe to bring back the passenger pigeon were discussed, as well as other broader efforts of prominent organizations to derive a list of good candidates for de-extinction. Concerns were mentioned at the end of the case as invasiveness, habitat loss, and new vectors for disease transmission [39]. This case study was typed as “Unknown/uncontrollable” as such species have not been brought back to life and placed in the environment yet and if they were, the point would be for them to re-establish on their own thus the controllability would be outside of human control.

The fourth and final case described the use of highly engineered microbes that typically associate with plants in order to impart nitrogen-fixation or other abilities on certain plants [40]. The benefits to agriculture and the environment were mentioned, along with concerns about releasing these bacteria into food crops. This case study was typed as “Known/Uncontrollable” as such genetically engineered microbes, such as *Rhizobium*, have been released in the past, the bacteria would already live in soil environments, yet they would be released on a wide-scale in food crops so their spread and travel could not be tightly controlled.

We hypothesized that the de-extinction case would have the overall highest level of concern (sum of all SRES scores) given that it is both uncontrollable and unknown (relative to the other cases). From the grid, biomining would be expected to be lowest according to the SRES (Controllable and more Known).

### SME Scores of Cases for Societal Risk Evaluation Scheme

**Fig 2:**
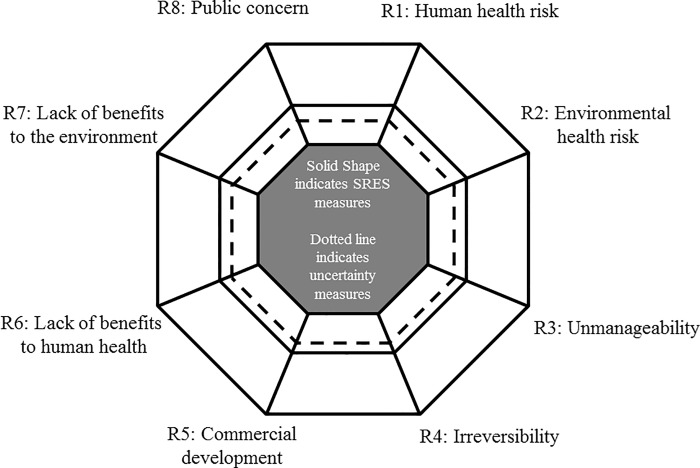
Example of SRES Octagonal Plot of Risk and Uncertainty

**Fig 3:**
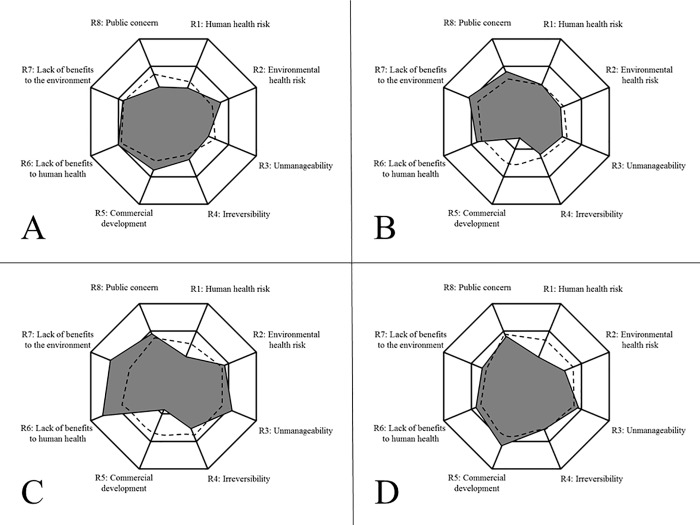
SRES Plots of Synthetic Biology Applications. (A) SRES Plot of Biomining Risk and Uncertainty. (B) SRES Plot of Cyberplasm Risk and Uncertainty. (C) SRES Plot of De-extinction Risk and Uncertainty. (D) SRES Plot of Plant Microbes Risk and Uncertainty.

The SRES was developed based on the interview data and the high-level themes that experts identified for synthetic biology cases to broaden the RES framework from Latxague et al. (2007) and Suffert et al (2009) and apply it in the specific context of synthetic biology. In addition to the 8-criteria scheme diagrammed in Fig 2, the mean risk (*R*) and uncertainty (*U*) scores were calculated. These scores also fall upon the 0–9 integer scale for ease of use by stakeholders and decision makers. It is again worth noting that we operationalized uncertainty as a negative degree of confidence regarding expert knowledge of all dimensions for each application under investigation. The descriptive statistics for the 8-factor scheme are provided below (Table 4). Fig 3A–3D shows the results for each case study.

**Table 4:** Mean Scores of SRES Evaluation Criteria

	Synthetic Biology Application							
	Biomining		Cyberplasm		De-extinction		Plant Microbes	
	M	SD	M	SD	M	SD	M	SD
R1: Human health risk	3.63	(1.7)	4.06	(1.7)	3.60	(1.7)	3.06	(1.7)
*Expert uncertainty of measure*	*4*.*42*	*(2*.*3)*	*4*.*00*	*(2*.*5)*	4.60	*(2*.*5)*	*5*.*11*	*(2*.*2)*
R2: Environmental health risk	5.09	(1.6)	3.46	(1.8)	5.71	(1.6)	4.37	(1.4)
*Expert uncertainty of measure*	*3*.*97*	*(2*.*3)*	*4*.*14*	*(2*.*7)*	5.23	*(2*.*1)*	*5*.*00*	*(2*.*3)*
R3: Unmanageability	4.63	(1.7)	5.17	(1.8)	6.34	(2.1)	5.66	(2.0)
*Expert uncertainty of measure*	*5*.*03*	*(2*.*3)*	*5*.*43*	*(2*.*6)*	4.60	*(1*.*9)*	*4*.*57*	*(2*.*2)*
R4: Irreversibility	4.23	(1.6)	3.71	(1.7)	4.83	(2.0)	4.77	(1.6)
*Expert uncertainty of measure*	*3*.*66*	*(2*.*4)*	*3*.*86*	*(2*.*8)*	5.29	*(2*.*0)*	*4*.*77*	*(2*.*0)*
R5: Commercial development	5.34	(1.9)	2.37	(1.8)	2.77	(2.2)	6.37	(1.7)
*Expert uncertainty of measure*	*4*.*34*	*(2*.*3)*	*4*.*91*	*(2*.*5)*	5.37	*(2*.*4)*	*5*.*57*	*(2*.*1)*
R6: Lack of benefits to human health	6.00	(2.0)	5.69	(2.0)	7.63	(1.7)	5.40	(2.1)
*Expert uncertainty of measure*	*5*.*71*	*(1*.*9)*	*5*.*37*	*(2*.*3)*	5.37	*(2*.*7)*	*4*.*40*	*(1*.*9)*
R7: Lack of benefits to the environment	5.40	(1.8)	6.06	(1.7)	6.77	(2.0)	4.86	(2.0)
*Expert uncertainty of measure*	*5*.*40*	*(2*.*2)*	*5*.*31*	*(2*.*4)*	4.66	*(2*.*1)*	*4*.*57*	*(2*.*0)*
R8: Public concern	3.97	(2.1)	5.80	(2.0)	5.89	(2.1)	5.77	(1.8)
*Expert uncertainty of measure*	*5*.*29*	*(2*.*0)*	*4*.*77*	*(2*.*2)*	5.57	*(1*.*8)*	*5*.*86*	*(2*.*1)*
Mean Risk Score (*R*)	4.79		4.54		5.44		5.03	
*Mean Uncertainty Score (U)*	*4*.*73*		*4*.*72*		*5*.*09*		*4*.*98*	

The data reported in each SRES model can be used to various capacities. First, the models allow for granular assessment of each criterion of the SRES in terms of both its risk profile as well as expert uncertainty of current understanding of that given criterion. For example, in the case of cyberplasm, there is a wide margin between the expert panel’s estimate of the likelihood of commercial development in the next 15 years (M = 2.37, SD = 1.8) and a much higher degree of uncertainty about this estimate (M = 4.91, SD = 2.5). This *prima facie* understanding of expert judgment signals a need for improved knowledge of the likely of near-term commercialization of cyberplasm, especially as UOA and governance initiatives are considered. Also, the mean risk (*R*) and uncertainty (*U*) scores allow for more robust cross-case comparison to note current expert views on the dimensions assessed within the SRES that may better situate future research and identify information and data needs.

The expert rating of the scores for biomining held a middling risk score (*R* = 4.79) and a similar uncertainty score (*U* = 4.73). Biomining also had distinguishing features of low public concern (M = 3.97, SD = 2.1) and low irreversibility (M = 4.23, SD = 1.6) (or high reversibility) of the application, higher degree of manageability (M = 4.63, SD = 1.7) (aka low unmanageability as shown in Fig 3A), and relatively higher ratings of the lack of benefits to human health (M = 6.00, SD = 2.0) and the environment (M = 5.4, SD = 1.8) when compared to the other case studies.

The cyberplasm case study was the lowest in its risk and uncertainty scores (*R* = 4.54, U = 4.72) and had distinguishing features of higher ratings for the lack of benefits to human health (M = 5.69, SD = 2.0) and the environment (M = 6.06, SD = 1.7), and high uncertainty of expert judgement regarding these items where M = 5.37, SD = 2.3, and M = 5.31, SD = 2.4 respectively. Most strikingly, experts also noted a very low degree of likelihood for commercial development of cyberplasm (M = 2.37, SD = 1.8) but noted much higher uncertainty about this criterion (M = 4.91, SD = 2.5). Many panel members questioned the utility and potential use of the SB application. One panel member responded to the utility and feasibility of the technology by saying, “it’s an interesting conceptual idea, but I don’t know where it is supposed to go… they’ve got a great little device they don’t really have a theory about how [we would use it], why we would need it, and where it would go.” Others noted that the low likelihood of commercial development and speculative nature of the application’s potential use may hinder productive dialogue regarding risks and benefits of the application and increase the uncertainty of forecasting its degree of manageability and irreversibility. The severely low likelihood of commercial development is also a likely factor for why overall risk of cyberplasm was lower than the other three applications.

The de-extinction case had the highest risk (*R* = 5.44) and uncertainty (*U* = 5.09) scores of the four cases. The expert panel noted that the SB application would not benefit to environment (M = 6.77, SD = 2.0) or human health (M = 7.63, SD = 1.7) and that it may pose significant risks to the environment (M = 5.71, SD = 1.6). They also were highly uncertain about the potential human health risks (M = 4.6, SD = 2.5) and the likelihood for commercial development of the application (M = 5.37, SD = 2.4) but held low ratings on each risk measure (M = 3.6, SD = 1.7; M = 2.77, SD = 2.2). In this case the panel was consistently uncertain in their estimates indicating a strong need to improve information and data on the risks, benefits, and societal impacts of de-extinction. One panel member highlighted some of insufficiencies in the data noting, “we can’t currently really assess the impacts of introducing species, or even what happens when a species is eliminated and how that impacts the larger ecosystem… you’re going to need to look at potential viruses and other diseases that may be reintroduced with that species, and also the impact that that species is going to have in the interactions with the current status of that ecosystem wherever it is you may make the reintroduction.”

The final case, microbes for nitrogen-fixing in non-legumes had the second highest risk and uncertainty score, (*R* = 5.03, U = 4.98) and a much higher likelihood of commercial development than the other cases (M = 6.37, SD = 1.7). It also had higher ratings of uncertainty regarding risks to human health (M = 5.11, SD = 2.2) and the environment (M = 5.00, SD = 2.3), and noted a higher estimate regarding the unmanageability of the application once used *in situ* (M = 5.66, SD = 2.0). Coupled with the apparently imminent development of the application, this may demonstrate a pressing need for improved risk analyses in the short term. One panel member succinctly highlighted such questions and concerns of the impacts of this application:

“you’re talking about a wide distribution, you’re talking about an open system, you’re talking about the potential of these microbes to interact with the rest of the soil, and the rest of the environment, and the plants, and it’s extraordinarily unclear what might happen… And then from there of course you don’t know how that’s going to change the planet. Are we humans going to be consuming part of those or entire microbial ecosystems with the plant whole? How are animals being fed off those as well, or graze on these plants or crops, or not? How they are going to be affected and so on and so forth. So, extraordinarily broad potential for impact for the general ecosphere.”

In looking at the individual element of the SRES framework, our typing of the cases appeared justified. The two case studies with less controllability were rated the highest for “Overall Risk” and “Uncertainty” according to the SRES framework (Table 4). Familiarity, rated under the “likelihood of commercial development” was not as much a predictor of these measures, especially for biomining. When scores of irreversibility and manageability are added, plant microbes and de-extinction came out higher than biomining and cyberplasm, lending credence to their “uncontrollability”. The typing of familiarity also seemed to be valid in that “likelihood of commercial development” was rated lower for both de-extinction and cyberplasm, lending the notion that these technologies will not be as familiar as biomining and plant microbes. In this sense it seems that for these SB cases, controllability may edge out familiarity regarding their importance in the EHS risk ratings, and that during such early-to-mid stage development, we should consider prioritizing these dimensions of associated risks over others when assessing governance needs. Such notions were corroborated by the expert panel and identified in subsequent analysis of risk governance themes from the qualitative interviews detailed in the coming section.

Also noted of this data set are the comparably large standard deviations along with mean scores. Standard deviation was typically higher for measures of uncertainty than for the primary 8 SRES criteria. This is a likely function of the novelty of the chosen case studies, as there is very little data on the potential societal risks of these SB applications, and of the multidisciplinary background of the panel members themselves, as some experts would be unlikely to report confidence in areas outside of their particular domain (although this may not always be the case). Expert elicitation for emerging technological risks often shows a wide range of expert scores (see for example [34]). Furthermore, the expert group was selected to represent a range of views and biases on precaution versus promotion of technology in governance systems. While a granular look at the responses of individual SME groups categorized by discipline or affiliation would be of value in the future, we feel that the current sample size and multiple expertise areas of the individual experts (see Table 1) would not allot for adequate comparability of different groups’ scores. Future study may compare SME groups to note differences in risk and uncertainty ratings.

As we have shown in this section, the SRES framework can be used in a numerical screening process to identify areas of highest concern or uncertainty, thus addressing our first question on “practical decision making”. To address our second set of research questions on “methods and process”, we describe how SRES can be integrated into deliberative models that account for multiple types of impacts. We illustrate how interviews can provide more detail on key areas identified from a SRES screening process, and we highlight examples of how the SRES can be used for prioritizing data, dialogue, or information needs to inform decision making.

### Dominant Risk Governance Themes from Expert Interviews

**Fig 4:**
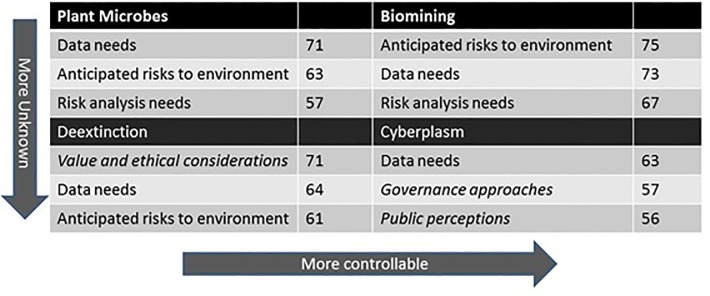
Qualitative Interview Themes

Interview data that were collected in the first round of the Delphi study, and subsequently thematically coded, are useful for understanding expert ratings of the risk and uncertainty for each technology. In Fig 4, the top three interview themes for each case study are presented (as normalized by total themes per case study). From this analysis, it is interesting to note that the two case studies with the least familiarity prompted more mention of values, ethics, governance, and public engagement by the SMEs. Whereas, the cases with more familiarity (biomining and plant microbes) tended to prompt themes that fit more traditional risk analysis that focuses on health and environmental hazards and data needs. This finding supports the notion that familiarity may be more important than controllability for broader public conversations about factors lying outside traditional regulatory review. In contrast, controllability seemed more important for overall risk and uncertainty rankings according to the quantitative results of the SRES framework (Table 4). Thus, we found that the SRES, in combination with interviews, contributes to theory-building and the identification of features of SB applications that are important for overall risk rankings and priority areas.

Uncertainty, in various forms, was another important theme expressed by the experts during the open-ended interviews, with 66 total references. Uncertainty was expressed by the SMEs as containing elements of both “unknown-ness and unfamiliarity” and “uncontrollability” in their statements. Another form of uncertainty, that being the expert’s reflective accounts of their uncertainty of risk judgments is highlighted in the SRES models and serves to demonstrate the level to which experts provide uncertain judgments about the SB case studies. Here, higher levels of overall uncertainty represent the degree to which the expert group cautions their level of certainty regarding their solicited judgments about the 8 SRES measures. De-extinction and plant microbes had the overall highest level of uncertainty according to the SRES (Table 4), and it is interesting to note that these are the cases also typed as uncontrollable. We provide sample quotes below from the interviews for these two cases and the themes of uncertainty and controllability.

Regarding the potential use and benefit of de-extinction, many experts expressed the lack of control that is likely associated with the technology. One expert stated, “thinking about passenger pigeons, that actually is going to be, you know, if that works that’s a—that’s a massive introduction that spreads nationwide.” Experts also noted significant concerns of virus reintroduction when bring back extinct species stating that a host of little known “zoonotic diseases” could be reanimated as well as the target species. Another panel member noted the difficulty in characterization of potential risk outcomes stating the need for “a huge amount of data, I mean whether it is say the passenger pigeons, whether they had any diseases or something that they could currently spread to existing bird species, or even other types of animals out in the environment.” Such extreme data needs mirror de-extinction’s uncertainty ratings. In the context of plant microbes, expert statements also support high uncertainty related to the information regarding human health and the health and environmental risks for this technology. One expert stated that for plant microbes, “…there’s sort of the potential agricultural and health effects of given what’s left behind, or given how things are altered,—how will that potentially negatively impact living organisms, in particular humans and agriculturally important organisms like cattle, or pigs, or sheep, or whatever?” In regards to human health, one expert states, “—there’s going to have to be all sorts of human health talks, allergenicity type of testing data to ensure that worker exposure is okay, and also if it’s an environmental introduction… it of course has some exposure to the general population”.

### SRES Methodological Contributions to Anticipatory Governance

The SRES can be used as a screening, data prioritization, and dialogue tool in anticipatory governance of SB applications. There is a need to anticipate and prepare for SB applications before deployment, yet at these early stages, the risks, benefits, and broader societal concerns have not usually been evaluated. In a previous study, we describe a dynamic oversight approach designed to anticipate the impacts associated with emerging technologies prior to commercialization, so that we can better prepare for future decisions about whether or how to deploy them. Dynamic oversight proposes an institutional model with three advisory groups working together to screen categories of emerging products in order to identify concerns, hopes, and research needs for future decision making [41]. One of these groups would be an interagency government task force to employ regulatory and legal requirements; a second would be a stakeholder advisory group represented by multiple interests, sectors, and disciplines; and the third would include private citizens with local and specialized knowledge who would serve as regional representatives of larger, national-scale public deliberations. The three groups would periodically convene together, and each would have formal input into oversight and decision making. The SRES framework could be integrated into a model for anticipatory governance like dynamic oversight.

**Fig 5:**
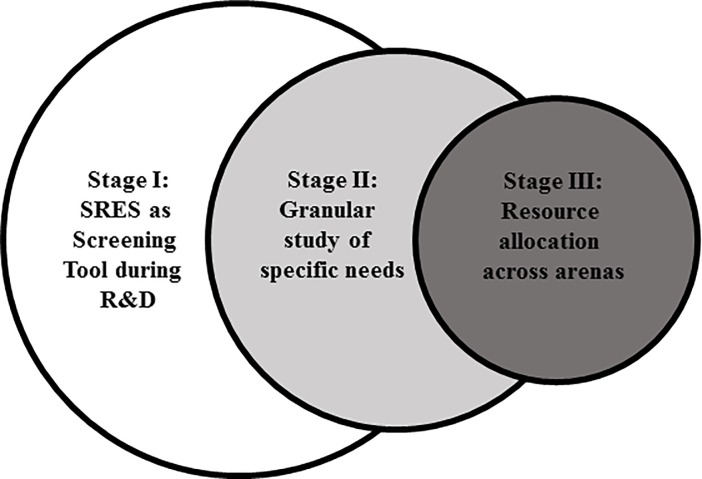
Proposed Timeline for use for the SRES

With multiple applications of SB coming down the development pipeline and in the face of limited capacity and resources for oversight, the SRES framework could be used as a tool to focus the work of these advisory groups on certain SB applications; for example, the ones that have the highest overall risk scores like de-extinction and plant microbes (Table 4), or on certain concerns within a specific SB application, for example, the SRES factors that have the highest rating or uncertainty. The visual models for these case studies can be scanned to identify areas of priority for gathering additional information, funding risk science, and hosting public engagement events (Fig 5).

For example, the groups involved in dynamic oversight might choose to focus on the case study with the highest overall SRES rating, de-extinction and focus on manageability, an area of high concern in the SRES for this case (Fig 3C). Funding could then be allocated to the development of risk management plans for introducing extinct species back into the wild. The SRES also allows for consideration of uncertainty levels to prioritize risk governance efforts. For example, the advisory groups in dynamic oversight might focus on the high degree of uncertainty about the human health impacts of using engineered plant microbes and commission research on mammalian exposure to plant material inoculated with the microbes.

Another way to use the SRES in the dynamic oversight model is in combination with the theory it supports. Controllability and familiarity seem to be important factors in upstream assessments of applications of SB. The visuals in Fig 3 can help to identify controllability through the “manageability” and “irreversibility” factors and familiarity with the “likelihood of commercialization” ratings. For SB applications of low controllability (e.g. open release) and high familiarity, our findings suggest that the traditional collection of health and environmental risk and benefit data is of high importance; whereas for cases of low familiarity, public engagement with value-based discussions may be more important. These examples illustrate just a few of the specific ways that the SRES can be used to better inform anticipatory governance processes.

### Limitations

Like all novel studies and theoretical works, this project has its limitations. First, the Policy Delphi process itself has its limitations. As outlined by Franklin and Hart (2007) [42], appropriate panel selection is crucial for the success of a Policy Delphi study. Of vital concern is the validity and relevance of the expertise area given the goals and objectives of the project. As this project was founded to identify benchmark data and information needs regarding potential health, environmental, and social risks of near-market SB applications we chose a multidisciplinary panel with a variety of subject-matter expertise. This has helped us to achieve goals of identifying a plethora of challenges and opportunities across disciplines, but we also recognize that it may provide less depth of findings in any one area that could be maximized through purposive sampling of more targeted expertise areas (e.g. a panel of only ecologists) should more granular research questions have been initially posed to guide the project. Another limitation of Policy Delphi studies may be the large time commitment given by panellists who spent many hours and even days travelling at times, to participate in the lengthy project like this one. As Franklin and Hart note of their own Policy Delphi study, “[i]t can be argued that only those individuals with strong opinions about the topic were interested in participating in such an intensive process” (p. 242). The same may certainly be true of our study as well, and we would also note that the panel was comprised of younger scholars who may be more enthusiastic to work in this novel multidisciplinary arena. Finally, as is the case of all Policy Delphi studies, the data analysis process is subjective. Researchers develop the initial questionnaire to guide inquiry, but then subsequent rounds of study involve interpreting qualitative interview data, refining it into the next round’s questioning, and condensing such information into quantitative metrics. As such, replication of study findings using the collected data would also be subject to researcher input. Although such limitations exist, we feel that the positive gains of this novel approach may be of substantial use for future researchers in this area.

## Conclusions

This paper provides aggregated multidimensional, societal risk profiles of developing SB applications. A new SRES framework is presented to address the broad research questions that asked about how societal impacts of SB technologies be incorporated with traditional EHS estimates to better inform decision-makers, and how uncertainty of information, be incorporated into risk and benefit estimates of SB technologies. The SRES framework also provides new answers to research questions that ask how we can improve practical decision making as it provides a theoretically-based novel method and process for assessing current expert understanding of real-world cases that can advance anticipatory risk governance initiatives of SB technologies under high uncertainty.

The SRES framework incorporates expert-stakeholder perceptions of risk and benefit measures along with measures of public concern, likelihood of development, manageability and reversibility, and uncertainty in order to provide a robust portrayal of profiles for each case that can be used to improve governance dialog and promote prioritization of future research and data collection. As such, it extends previous work on MCDA and emerging technologies by focusing on criteria important for deployment of SB applications into the environment and by using a mixed-method policy Delphi methodology [16]. The SRES can be used as a tool for helping to prepare governance systems for SB applications while they are early in development through the direction funding for research or public engagement. Under the idea of preventative governance [14], it can also serve to help policy-makers decide whether to encourage or prevent deployment of these applications based on comparison with existing or alternative technologies.

The SRES framework may serve to expand traditional risk analysis in a meaningful way to provide a more comprehensive picture of potential harms and concerns. We submit that this method is well-suited for anticipatory risk governance and can be used in the face of significant uncertainties. In these situations, the goal is not to predict but rather to prepare. SRES could provide a useful and comprehensive screening tool to highlight general areas where more information and dialogue are needed.

The visual models produced from this exercise are useful for identification of expert group perceptions of individual risk factors, overall perceptions of a developing technology, as well as comparative assessment of how certain experts feel about their risk ratings. This becomes even more informative when multiple cases are used in conjunction with one another. Such cross-case comparison can highlight significant information needs, and may be useful for prioritizing future research agendas. Furthermore, the ability to summarize this abundance of data into simple visual graphics increases the accessibility of such information and serves as a powerful, yet simple, analytic tool that enable accelerated heuristic processes of information assimilation. Under limits of budgets and time, the SRES presents a quick method to rank factors and uncertainties so that more data and information can be obtained in areas that appear to be most important. The framework can then be adapted as necessary to update relevant factors as the product is developed.

We propose that the SRES first be deployed at early stages of product development, preferably prior to commercial investment. At this stage it may contribute significantly to highlight data and information needs, and may highlight pressing areas of concern as noted from the case studies we presented here. Subsequent dialogues and studies of specific needs can then focus on these more granular areas of concern. This new information may then form a greater basis of knowledge and data that may serve as a resource for future decision-making across multiple arenas including product development, risk management, and governance and risk communication. In this sense, the SRES may be a good mechanism for establishing baseline understanding of a case study from which subsequent work can be prioritized. It complements previously described top-down approaches to decision analysis in which technical data to assess the risk is combined with decision criteria and value judgments expressed by to prioritize further research that will reduce the most uncertainty [43].

The SRES model may also be integrated into wider stakeholder deliberation and assessment models. For example, the International Risk Governance Council (IRGC) proposes that when there is high uncertainty and ambiguity, a wider range of stakeholders and interested citizens should be involved in deliberating about risk governance information needs and policy choices [44]. The SRES framework could be used as a screening tool for focusing on applications of emerging technologies that are of most concern to stakeholders and interested and affected citizens in such deliberative processes. In the IRGC’s recent report on guidelines for emerging risks and governance, the SRES can help to fill necessary capabilities in enhancing proactive thinking to identify future threats and opportunities; prioritising investments in certain key emerging issues according to their potential impact; and fostering internal communication and building a forward-looking culture [45]. SRES focuses on future applications of technology and provides a way to begin to assess them under conditions of high uncertainty. It also uses a broader framing of risk that integrates and respects not only toxicological definitions of risk, but also technological, economic, and social ways of assessing risk.

The SRES framework aligns well to conceptions of post-normal science, and employs scenario-based multi-criteria risk evaluations in order to rapidly assess cases where “facts are uncertain, values in dispute, stakes high and decisions urgent” [46]. Such work aligns to the goals of responsible research and innovation to “effectively evaluate both outcomes and options in terms of societal needs and moral values,” in order to establish a “collective, inclusive, and system-wide approach” to the design and development of new technologies [47]. The SRES approach may also be furthered by integrating expert-derived data into other multi-criteria decision analytic frameworks [15]. Such MCDA frameworks can assist policymakers with assessing multiple alternative policy options regarding the anticipated governance of SB applications. Use of robust expert-derived data like those presented in this paper could serve as a vital component of decision-making and regulation of future SB technologies.

Appropriate governance mechanisms have the potential to minimize harm and improve the chances for broad public benefits from SB, while respecting a range of societal values. In summary, we have developed an approach to better prepare for future governance of SB. We “unpack” the broad field of synthetic biology by using individual applications of SB and identifying important areas of attention or for information collection and dialogue Using SRES with subsequent dialogue approaches, can help to set priorities for governance and the focus can shift to the domains of SB needing the most attention. Resources for risk-relevant data collection, organizational and legislative readiness for oversight, and public and stakeholder engagement can be directed towards these areas. This approach is especially important given the pace of technological development and the need for governance to match that pace under conditions of scarce resources.
